# THz near-field spectral encoding imaging using a rainbow metasurface

**DOI:** 10.1038/srep14403

**Published:** 2015-09-24

**Authors:** Kanghee Lee, Hyun Joo Choi, Jaehyeon Son, Hyun-Sung Park, Jaewook Ahn, Bumki Min

**Affiliations:** 1 Department of Mechanical Engineering, Korea Advanced Institute of Science and Technology (KAIST), Daejeon 305-751, Republic of Korea; 2 Department of Physics, Korea Advanced Institute of Science and Technology (KAIST), Daejeon 305-751, Republic of Korea

## Abstract

We demonstrate a fast image acquisition technique in the terahertz range via spectral encoding using a metasurface. The metasurface is composed of spatially varying units of mesh filters that exhibit bandpass features. Each mesh filter is arranged such that the centre frequencies of the mesh filters are proportional to their position within the metasurface, similar to a rainbow. For imaging, the object is placed in front of the rainbow metasurface, and the image is reconstructed by measuring the transmitted broadband THz pulses through both the metasurface and the object. The 1D image information regarding the object is linearly mapped into the spectrum of the transmitted wave of the rainbow metasurface. Thus, 2D images can be successfully reconstructed using simple 1D data acquisition processes.

The terahertz (THz) region usually includes frequencies ranging between 0.1 THz and 10 THz, and this frequency region has attracted significant interest recently^1^,^2^. THz imaging is one of the most important research topics in the applications of THz waves due to its potential in various fields, such as biomedical applications and non-destructive inspections^3^,^4^,^5^,^6^. Because conventional THz imaging systems involve raster scanning an object through the beam focus^3^, the long image acquisition time is a major limiting factor in real applications. To build a high-speed imaging system, one solution is spectral encoding techniques, such as nuclear magnetic resonance imaging^7^, endoscoping using spectral encoding^8^, and serial time-encoded amplified imaging^9^. In these imaging methods, data acquisition is simple and fast because spatial image information is encrypted in the spectrum. THz imaging systems based on THz time domain spectroscopy (THz-TDS) have great advantages in the utilization of spectral encoding techniques because single-cycle THz pulses used in THz-TDS have an extremely broad bandwidth as large as their carrier frequency. Several THz imaging systems have been developed based on spectral encoding, such as tomography using a Fresnel lens^10^ and single pixel coherent diffraction imaging constructed with a coherent optical computer^11^ or a slanted phase retarder^12^.

A metasurface is composed of a two-dimensional array of subwavelength-scale resonators, and it is expected to enable numerous optical functionalities via wavefront engineering^13^,^14^,^15^. In this work, we devise a space-frequency converting metasurface for THz spectral encoding imaging. The devised metasurface is composed of mesh filters of various sizes, and these mesh filters are spatially arranged such that their pass frequencies are proportional to their one-directional position on the metasurface, similar to a rainbow. For whole two-dimensional imaging, two different types of imaging systems are constructed using rotation and translation scan processes. In our first imaging system using the rotation scan process, the imaging target is placed very close to the front of the rainbow metasurface, and broadband THz pulses are transmitted through both the metasurface and the target. In this configuration, the collected spectrum of the transmitted THz waves represents an angular projection image of the object; 2D imaging is achieved with sufficient measurements at different rotating angles. In our second imaging system using the translation scan process, an additional narrow slit is inserted between the rainbow metasurface and the target. Then, the target is scanned to acquire the image information in the scanning direction while information in the other direction is obtained by the spectrum analysis of the transmitted THz waves.

Our imaging experiment was performed using a typical THz-TDS system^16^ based on a Ti:sapphire oscillator laser with a repetition rate of 80 MHz. The average power and centre wavelength of the laser were 750 mW and 810 nm, respectively. To generate and detect the THz waves, we used a commercially available large area photoconductive antenna^17^ and utilized an electro-optic sampling technique with a 2 mm thick <110>-oriented ZnTe crystal. The designed rainbow metasurface and transmission properties of its constituent mesh filters are shown in Fig. 1. The rainbow metasurface is composed of 200 nm-thick gold patterned onto 1.8 μm-thick polyimide and was made by a conventional fabrication technique via photolithography^18^. Complementary cross-type mesh filters^19^ with varying geometrical parameters (width *d*, length *L*, and lattice constant *a*) were spatially arranged to construct the rainbow metasurface, as shown in Fig. 1(a). Because each mesh filter has a unique passing frequency band, a one-to-one correspondence between the spatial and spectral information can be achieved. To control the passing frequency, the overall size of the mesh filters was adjusted while maintaining the geometric scaling factors, such as *a* = 1.5 and *L* = 30*d*. To determine the sizes of the constituent mesh filters, the geometrical parameters of the five mesh filters having centre frequencies, ω_c_, of 0.2, 0.7, 1.1, 1.6, and 2.0 THz were first obtained by finite-difference time-domain (FDTD) simulation. These transmission spectra are shown in Fig. 1(b). Then, a fitted relation between the size and centre frequency was established as 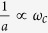 based on the FDTD simulation results, as shown in Fig. 1(c). According to this fitted curve, the size of a mesh filter at a given position, *y*, must satisfy the condition *y* = *αω*_*c*_, where *α* is a proportionality constant. The fabricated rainbow metasurface has a footprint of 20 × 20 mm^2^, and the centre frequencies of the mesh filters ranged from 0.2 THz to 2.0 THz. Therefore, the spectral encoding proportionality constant *α* was 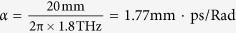 for the rainbow metasurface.

The rotation scanning imaging experiment is schematically illustrated in Fig. 2(a). In this setup, an image target shown in Fig. 2(b) is placed in front of the rainbow metasurface. Both the target and the metasurface are placed in the collimated THz beam region, and the collimated THz beam width is large enough to envelop them. The measured spectral amplitude *V*(*ω*, *θ*) at the rotation angle of *θ* can be expressed as


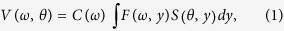


where *C*(*ω*) represents the spectral scaling factor induced from the spectral field profiles of the incident THz wave and the spectral dependence of focusing on the detector plane^20^,^21^, *F*(*ω*, *y*) is the amplitude transmission of the metasurface at *y*, *S*(*θ*, *y*) is the projection of the image in the *θ* direction given as 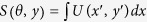, where 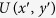 is the image information, and *x*′ = *x* cos *θ* + *y* sin *θ*, *y*′ = −*x* sin *θ* + *y* cos *θ*^22^. If we assume that the bandwidth of the mesh filters in the rainbow metasurface is very narrow, or *F*(*ω*, *y*) = *δ*(*y* − *αω*), where *δ*(*y*) is the Dirac delta function, then the rainbow metasurface encodes the projection of the image to the spectrum as 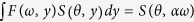. Therefore, with a sufficient number of *V*(*ω*, *θ*) measurements at angles between 0 and 180 degrees with equal angular spacing, the image can be reconstructed from


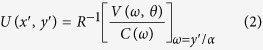


where *R*^−1^ denotes the inverse Radon transformation^22^. In our experiment, we measured 30 transmitted THz waveforms by rotating the target along the optical axis from *θ* = 0° to 174° with an angular spacing of 6°. The measured time domain signals and their corresponding spectra are shown in Fig. 2(c,d). The spectral scaling factor *C*(*ω*) is measured by a control experiment without the target, and the image is reconstructed based on Eq. (2), as shown in Fig. 2(e).

In real experiments, mesh filters making up the rainbow metasurface have a bandwidth that is characterized by a quality factor, 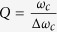; therefore, the amplitude transmission of the metasurface cannot be approximated as the Dirac delta function, i.e., 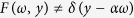. Therefore, the measured spectral amplitude does not encode spatial information of the object exactly, and the reconstructed image is blurred compared to the original target information. For more quantitative analysis, we perform numerical simulations on the reconstructed image using filters with a Lorentzian lineshape and various quality factors, *Q*. An image phantom is shown in Fig. 3(a) and reconstructed images using filters having *Q* = 4.5, 9, and 18 are shown in Fig. 3(b–d), respectively. According to our FDTD simulation, the quality factor, *Q*, of the mesh filters is approximately 9; therefore, the reconstructed image shown in Fig. 3(c) is most similar to the reconstructed image from our experiment. As shown in Fig. 3(b–d), a simulation result with higher *Q* mesh filters has higher resolution. The image resolution may also be influenced by the sizes of the mesh filters and the distance 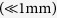 between the rainbow metasurface and the target object, but, in our imaging system, those are not major causes of the blur.

Our second imaging technique used translational scanning and is illustrated in Fig. 4. In this experiment, a thin aluminium slit of width (*w*) 0.6 mm, is placed in front of the rainbow metasurface, and the image target is horizontally scanned in the *x-*direction as shown in Fig. 4(a). We used the same target as in the imaging experiment using a rotational scan, and we obtained 60 temporal waveforms using 15 mm translational scans with 0.25 mm spacing, as shown in Fig. 4(b). Figure 4(c) shows the corresponding spectral amplitude, *V*(*ω*, *x*). Because the slit width *w* is small enough compared to the scale of the target, *V*(*ω*, *x*) can be represented as


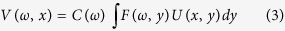


where *C*(*ω*), *F*(*ω*, *y*), and *U*(*x*, *y*) represent the spectral scaling factor, the amplitude transmission and the image information, respectively. Using the narrow filter assumption, 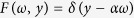, a set of 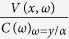 directly represents the image, *U*(*x*, *y*). The reconstructed image using this principle is shown in Fig. 4(d). In translational scan imaging, the vertical resolution of the image is determined by the quality factor of the mesh filters, *Q*, like in the rotational scan imaging. Conversely, the horizontal resolution of the image is determined by the slit width. Compared to rotation scan imaging, translation scan imaging has a much lower signal-to-noise ratio because the transmitted waves are blocked by the narrow slit in front of the rainbow metasurface. However, a precise alignment is not necessary in this geometry, whereas the target should be rotated along the optical axis in rotational scan imaging.

The main limitation of our current imaging system is the low quality factors of the mesh filters used to create the rainbow metasurface. Thus, spectral information produces blurred images. To overcome this, thicker mesh filters^23^ or other metal structures having higher quality factors^24^,^25^ could be used to compose a better rainbow metasurface. By using those structures known to have quality factors on the scale of tens or even a few hundreds, the imaging resolution can be significantly improved from that reported in this work.

In conclusion, we propose a novel image acquisition system that uses spectral encoding in a rainbow metasurface in the THz range. The rainbow metasurface is composed of sequentially arranged mesh filters of varying size and converts spatial image information into spectral information by attaching it to an image target. From an analysis of the measured spectral information, 1D image information is automatically acquired. Thus, the number of measurements required for a 2D imaging process can be drastically reduced. We demonstrated two different imaging methods using 1D rotational and translational scans, and reliable 2D images were reconstructed in both methods.

## Additional Information

**How to cite this article**: Lee, K. *et al.* THz near-field spectral encoding imaging using a rainbow metasurface. *Sci. Rep.* **5**, 14403; doi: 10.1038/srep14403 (2015).

## Figures and Tables

**Figure 1:**
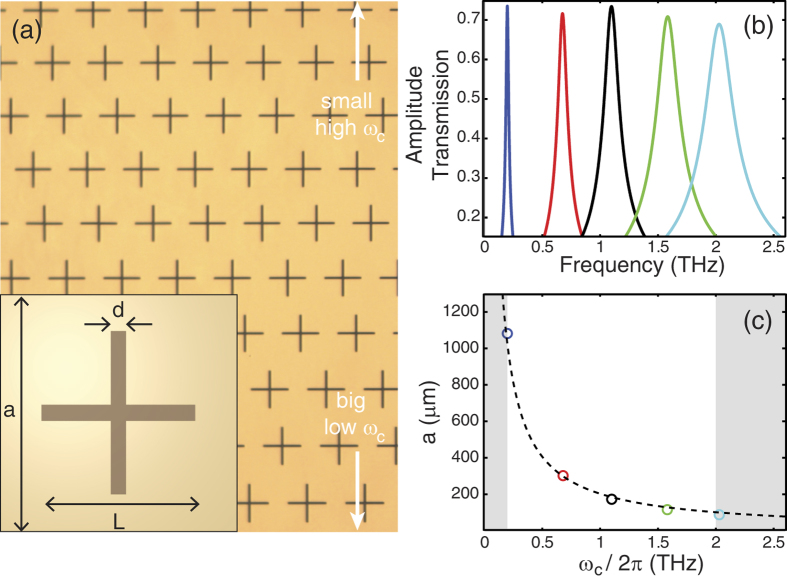
Rainbow metasurface. (**a**) Microscopic image of the rainbow metasurface. The inset shows our definitions of the geometrical parameters (width d, length L, and lattice constant a) for the mesh filters. (**b**) FDTD simulation results of the transmission of the mesh filters having centre frequencies at 0.2, 0.7, 1.1, 1.6 and 2.0 THz, respectively. (**c**) Relationship between the sizes and the centre frequencies of the mesh filters. Circles indicate FDTD results, and the dashed line indicates a fitted curve, 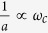. The white region denotes the frequency range used in the rainbow metasurface.

**Figure 2:**
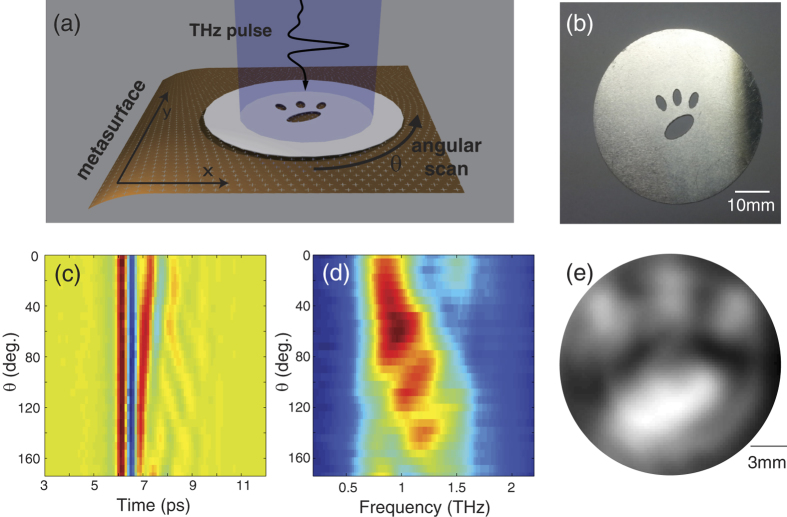
Imaging results in the rotational scan measurements. (**a**) Schematic drawing of the measurement. The scale of the mesh filters is exaggerated to illustrate their geometrical features. (**b**) Target object, a representation of a feline paw. (**c**) Measured THz temporal field profiles presented as a function of the object angle. (**d**) Corresponding THz spectra. (**e**) Reconstructed image using the inverse Radon transform. The scale bar is calculated from *α*.

**Figure 3:**
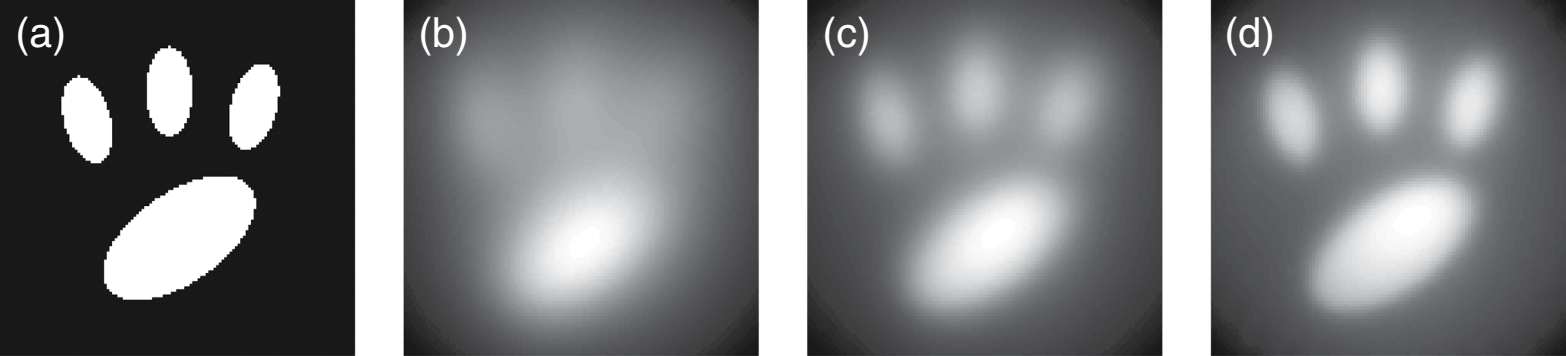
Simulation results of reconstructed images from the rotational scan measurements. (**a**) Image phantom. (**b–d**) Reconstructed images with the rainbow metasurface composed of mesh filters of different quality factors (**b**) Q = 4.5, (**c**) Q = 9, (**d**) Q = 18.

**Figure 4:**
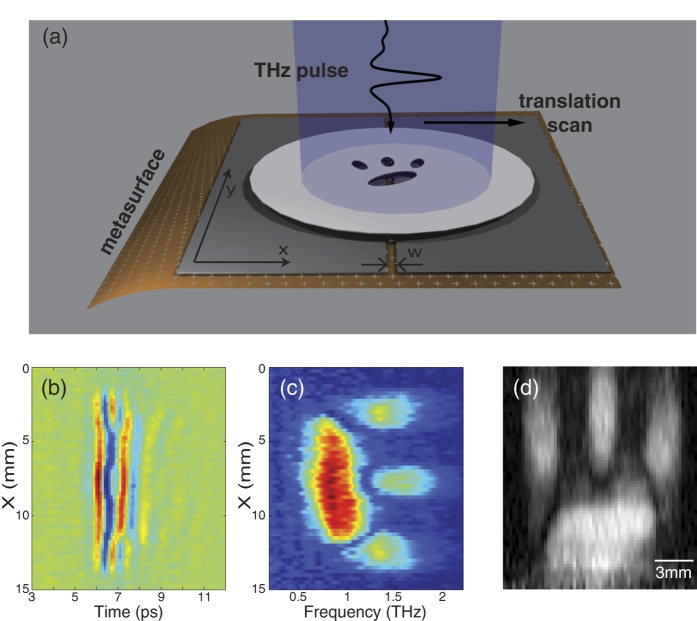
Imaging results in the translational scan measurements. (**a**) Schematic drawing of the measurement. A narrow slit of width *w* is inserted between the target and the metasurface. (**b**) Measured THz temporal field profiles presented as a function of the translational scan distance. (**c**) Corresponding THz spectra. (**d**) Reconstructed image.
